# Autochthonous Yeast from Pork and Game Meat Fermented Sausages for Application in Meat Protection and Aroma Developing

**DOI:** 10.3390/ani10122340

**Published:** 2020-12-09

**Authors:** Beatriz García-Béjar, Daniel Sánchez-Carabias, Marina Alarcon, María Arévalo-Villena, Ana Briones

**Affiliations:** 1 Department of Analytical Chemistry and Food Technology, University of Castilla La Mancha, 13071 Ciudad Real, Spain; marina.ahernandez@uclm.es (M.A.); maria.arevalo@uclm.es (M.A.-V.); ana.briones@uclm.es (A.B.); 2 Regional Institute of Applied Scientific Investigation (IRICA), University of Castilla-La Mancha, 13071 Ciudad Real, Spain; daniel.sanchez14@alu.uclm.es

**Keywords:** pork fermented sausages, game meat fermented sausages, autochthonous yeast, antioxidant activity, biocontrol, aroma formation

## Abstract

**Simple Summary:**

Yeasts are microorganisms presented naturally in meat products microbiota, which carry out an essential role in the maturation process of fermented sausages. The maturation of this type of product could be better controlled by the addition of a starter culture or a mix of strains that could standardize the process in diverse factories. Moreover, it would be favorable to find cultures that, not only conduct the curing process, but also present an added value such as the meat product protection (antioxidant capability and biocontrol activity). Yeasts also have an important role in the meat product aroma development. Therefore, it would be interesting to select a pleasant-aroma forming strain in order to elaborate a future starter culture for fermented sausage maturation.

**Abstract:**

The wild yeast community was studied in fermented sausages from pork and game meat (deer and wild boar) during the maturation process from different curing rooms. Although the biotechnological importance of yeasts in the maturation process of pork sausages is known, there is a lack of information for sausage maturation involving game meat. A total of 123 yeasts were isolated and, by amplifying and sequencing of the ITS region, were classified in 14 species. *Debaryomyces hansenii*, *Kazachstania servazzii*, and *Wickerhamomyces anomalus* were isolated in both pork and game samples. The PCR-RAPD technique differentiated between 26 and 18 strains from pork and game meat sausages, respectively. The physicochemical parameters and their relationship with the yeast community were also studied. The antioxidant and anti-lipid peroxidation capability were analyzed and the 70% and 50% of the tested strains showed these abilities, respectively. Moreover, the biocontrol capability against mycotoxigenic molds was found in 19 strains, but better results were observed in game meat yeasts. On the other hand, almost 30% of strains produce a pleasant olfactory aroma, and volatile compounds associated with the yeast pathway metabolic during the maturation process have been characterized such as esters, aldehydes, fusel alcohols, etc. This study has allowed a better understanding of the biodiversity of this type of food, as well as selecting potential yeast strains for their future use as starters.

## 1. Introduction

Mediterranean countries have a long tradition in the production and consumption of pork products. Moreover, in the past years, an increase in the acceptance, demand, and production of game meat has been observed. Game meat fit into the consumers’ ideology of healthy and environmentally friendly meat when compared to domesticated animals and it can be considered an alternative to domesticated animals [1].

The fermentation process in these products increases their shelf-life and improves the sensorial characteristics. Lactic acid bacteria contribute to the acidification during fermentation, whereas the Gram-positive and catalase-positive cocci are related to the lipolytic and proteolytic reactions and color improvement. Furthermore, molds and yeasts have an influence during both the fermentation and ripening process [2].

The maturation steps favor yeast cultures growth due to the associated conditions (low humidity, high salt concentration, low water activity, and pH). Moreover, yeasts have a protective effect against oxygen, since they are considered as oxygen scavengers and can delay rancidity and improve the aroma due to their lipolytic and proteolytic activity. Peromingo et al. [3] found some strains that could inhibit the molds growth that developed in meat products. 

*Debaryomyces hansenii* is the predominant species in the maturation process due to its tolerance to sodium chloride and nitrate and can be found from the beginning to the end of the sausage maturation. Another genera such as *Pichia*, *Yarrowia*, *Candida*, *Rhodotorula*, *Cryptococcus*, *Saccharomyces*, and *Trichosporon* have been isolated from dry fermented sausages, as well [4]. Nevertheless, few studies have been carried out on yeast populations in game meat. 

The aim of the present study was to identify the autochthonous yeast and characterize the biotechnological properties in pork and game meat in fermented sausages from different maturation periods and curing rooms.

## 2. Materials and Methods 

### 2.1. Sampling and Yeast Isolation

Fermented sausages (by duplicate) of pork (P) and game (M) meats at middle (30 days) and the end (60 days) of the maturation period were taken. Five curing rooms A, B (pork meat) and C (deer meat), D and E (wild boar meat) were sampled. The factories elaborated their products in a traditional way and did not use starter cultures. A total of 20 samples were studied.

Game meat was obtained from the Extremadura region (C and D) and Castilla—La Mancha region (E), which are one of the Spanish’s better-known hunting areas. The composition of the fermented sausages from game meat was as follows: Factory C—fats 36.5 g, proteins 24.2 g, and carbohydrates 3.6 g; Factory D—fats 32.7 g, proteins 27.4 g, and carbohydrates 4.8 g; Factory D—fats 43.6 g, proteins 22.4 g, and carbohydrates 1.3 g.

The sausages were sliced, and 10 g were homogenized in peptone water and 0.1% Tween 80 using a Masticator (IUL instruments, Barcelona, Spain). Samples or their decimal dilutions were inoculated onto a Yeast Extract Peptone Dextrose (YPD) agar (10 g/L yeast extract, 20 g/L peptone, 20 g/L glucose, and 20 g/L agar) with added biphenyl (200 μg/mL), chloramphenicol (100 μg/mL), and ampicillin (100 μg/mL) in order to inhibit mold and bacterial growth and then incubated (30 °C/4–5 days). Between 10–15 colonies with different morphologies were chosen from each sample, but if that was not possible, all of the grown isolates were taken.

### 2.2. Physiochemical Analysis of Meat Products

The water activity (Aw) was measured at 23 °C using the CX-2 Water Activity System (Aqua Lab, Meter Food, München, Germany). The chloride analysis was carried out according to the Möhr method, and the pH value was determined using a Crison Basic 20 pH meter. Measurements were made in triplicate experiments at two maturation periods.

### 2.3. Yeast Identification by Molecular Techniques

#### 2.3.1. Species Level

The genetic species identification of yeasts was achieved by amplifying the entire ITS region with ITS1 (5’TCCGTAGGTGAACCTGCGG3’) and ITS4 (5’TCCTCCGCTTATTGATATGCC3’) primers (Condalab, Madrid, Spain) [5]. Amplification was carried out on a Perkin-Elmer 2400 thermal cycler. PCR conditions were an initial denaturation at 95 °C/5’ and 35 cycles with the subsequent conditions: 95 °C/1’ (denaturation), 55.5 °C/1’ (hybridization), 72 °C/1’30’’ (extension), and a final cycle at 72 °C/10’ (final extension). PCR products were separated by electrophoresis on a 2% agarose gel and were visualized by the gel Green (6×) (Biotium, California city, CA, USA) in a gel documentation system. Then, the amplified PCR products were sequenced using the same primers (Macrogen, Madrid, Spain). Once the sequences were obtained, the identity was searched in the BLAST database (GenBank, Bethesda, MD, USA). An accession number was assigned to each uploaded sequence to the GenBank database (MK034958-MK034966; MK034979; MK034982-MK034985; MK034989-MK034992; MK034994-MK034996).

#### 2.3.2. Strain Level

With the aim of identifying the strain diversity, the PCR-Random Amplification of Polymorphic DNA (RAPD) the M13 primer (5’GAGGGTGGCGGTTCT 3’) (Takara Shuzo Co., Otsu, Shiga, Japan) was used. In addition, the protocol proposed by Fernandez-Pacheco et al. [6] was used. After the reaction, the products were separated by electrophoresis on a 1.5% agarose gel and were visualized by the gel Green (6×) in a gel documentation system.

### 2.4. Antioxidant Capability of Yeast Strains

This property was evaluated by the 2,2-diphenyl-1-picrylhydrazyl (DPPH) (Merck, Darmstadt, Germany) analysis expressed as a Trolox (Merck, Darmstadt, Germany) equivalent. One milliliter (106 cells/mL) from ON (overnight) cultures in a YPD broth was mixed with 1 mL of DPPH solution (0.06 mM) and incubated at 30 °C/30 min under dark conditions [7]. The optical density at 517 nm was measured (UV-Vis V530 Jasco, Champaign, IL, USA). A Trolox standard curve ranging from 0.02 to 0.35 mM was prepared and the assays were carried out by triplicate experiments.

### 2.5. Anti-Lipid Peroxidation Capacity of Yeast Strains

The ability of the yeast strains to avoid the lipid peroxidation was studied. A thiobarbituric acid (TBA) (Merck, Darmstadt, Germany) assay was carried out based on the protocol proposed by Liu and Huang [8] with some modifications. The pellet from ON cultures in YPD were dried at 60 °C for 2 h. The assay was carried out, in triplicate experiments: 3.6 mL of the lecithin solution (1 mg/mL in PBS pH 7.4) and 0.4 mL of the ferrous sulphate solution (25 mmol/L) were mixed with 0.4 mL of the pellet solution (0.3 mg/L). For controls, the biomass was substituted by 0.4 mL of vitamin C (0.01 mol/L) (positive control) and by 0.4 mL of PBS (negative control). The systems were incubated in a bath for 15 min at 37 °C. After that, 1 mL of trichloroacetic acid (TCA, 20%) and 1 mL of TBA (0.8%) were added and the tubes were stood in boiling water for 15 min. The absorbance was measured at 532 nm (UV-Vis V530 Jasco, Champaign, IL, USA). 

The anti-lipid peroxidation was calculated by applying the following formula:

Inhibition % = ((Anc − As)/Anc) × 100

where Anc is the absorbance of the negative control and As is the absorbance of the strain tested.

### 2.6. Inhibition Assay for Mycotoxigenic Molds

All yeast strains were tested against three mycotoxin-forming molds: *Aspergillus parasiticus* (CECT 2689), *Fusarium graminearum* (CECT 20487), and *Penicillium crustosum* (UCLM 93V). Each mold inoculum (10^6^ spores/mL) was dropped and placed onto the middle of a YPD agar plate. Young cultures of yeasts (YPD broth at 30 °C during 24 h) were dropped (10^6^ cells/mL) on the same plate (three yeasts spotted on each plate) and incubated at 30 °C for at least 5 days. 

The growth inhibition (inhibition %) of the mold was observed by comparing the radius of the positive control (mold cultured alone on an agar plate) with one yeast culture.

### 2.7. Aroma Production Capability in Two Synthetic Media

To establish a qualitative estimation of the aromatic impact of each yeast strain, two different aroma-producing media were used according to Cano-Garcia et al. [9]. The media used was the Yeast Nitrogen Base (YNB) broth (Difco-BD, Madrid, Spain) with 2-methylbutanoic acid (100 ppm) plus ethanol (20,000 ppm) or methanol (20,000 ppm). They were inoculated with 106 cells/mL and incubated at 30 °C/10 days at 130 rpm.

#### 2.7.1. Sensorial Aroma Screening

The olfactory properties were evaluated by a group of ten trained tasters that identified pleasant (floral, sweet, and fruits) and unpleasant aroma descriptors (cheese, grassy, and sulphur). Media without yeasts were used as negative controls. The strains that showed a pleasant aroma and one strain with an unpleasant aroma were chosen to study the volatile compounds.

#### 2.7.2. Gas Chromatography (GC) Analysis

Volatile compounds were identified and quantified by the headspace/solid phase microextraction (HS-SPME) and gas chromatography coupled to mass spectrometry (GC-MS). A triple SPME fiber of DVB/CAR/PDMS 50/30 μm (conditioned at 270 °C/30 min), was exposed for 1 h and 30 min at 40 °C. In a capped vial, 50 µL of each sample were dispensed. The volatile compounds were transferred to the GC injector and desorbed (260 °C/5 min). The analysis was performed in a spitless mode (50 mL/min during 2 min) on a 6890 N Agilent gas chromatograph coupled to a 5973 N Agilent Mass Detector. The selected chromatography conditions were: A polar column (DB-WAX, 60 m × 0.25 mm id; 0.25 μm film thickness), helium as the carrier gas (1 mL/min), and oven temperature started at 40 °C/3 min, ramped at 3 °C/min to 150 °C and up to 220 °C (7 °C/min for 5 min). The MS operated with an electron energy of 70 eV using the electron impact mode, the ion source temperature was 230 °C, and the scanning was carried out from 45 to 550 amu. Compounds were identified by comparison with mass spectra and the linear retention index (RI) with spectral data from NBS75K and Wiley G 1035 libraries, and with the Kovats index calculated using a series of standard n-alkanes. The semi-quantitative analysis by MS was performed assuming a response factor equal to 1. The results were expressed as μg/mL of the sample. Two controls that contained ethanol or methanol without cells were also studied.

### 2.8. Statistical Analysis

In order to identify significant differences, a Student’s T-test and analysis of variance (ANOVA) followed by Duncan’s test (significance level at 5%, *p* < 0.05) were applied, using IBM SPSS for Windows v. 24.

## 3. Results and Discussion

### 3.1. Yeast Counts

**Figure 1:**
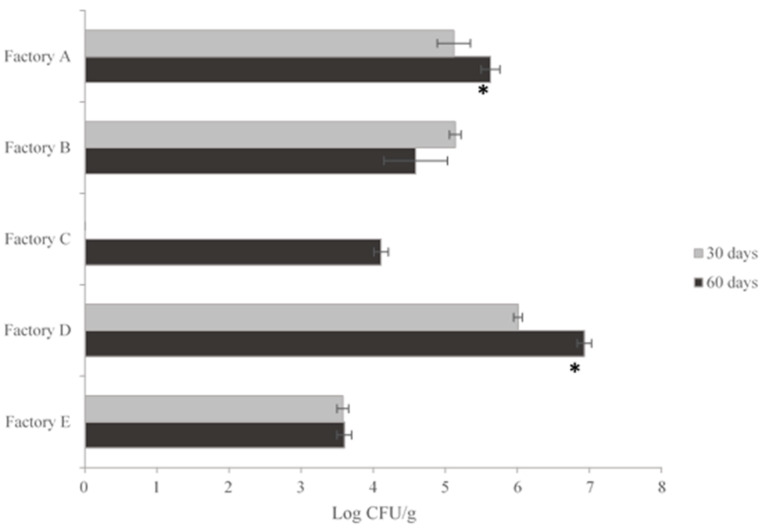
Yeast counts (log CFU/g) in pork and game meat fermented cured sausages at different ripening times. The * indicates significant differences, based on the Student’s T-test, (*p* ≤ 0.05) between cell counts at different curing times.

The values at different curing times are collected in Figure 1. They ranged between 3.6 CFU log (Factory E) and 6.9 CFU log (Factory D). The counts evolution in pork samples was different, therefore, in Factory A there was an increment on the counts with significant differences observed. However, in the samples from Factory B, the highest counts (5.1 CFU log) were detected after 30 days and a slight reduction occurred at the end of the maturation period (4.6 log units). The lowest cell counts were found in wild boar fermented sausages for both maturation times (Factory E) and no significant differences were detected neither in this factory nor in Factory C. Nevertheless, significant differences were observed between the yeast counts at different curing times in Factory D, as evidenced by the Student’s T-test. 

Cell counts at the end of the drying period provided an understanding of the yeast presence in these products and its association with the curing room environment. Mendonça et al. [2] and Cano-García et al. [9] found similar results to this study.

### 3.2. Physiochemical Analysis

The results were shown in Table 1. A slight reduction in the pH was observed, with the most marked differences found in pork meat. Wild boar products from factory E had the lowest final pH (5.02) and the highest value in the final product was found in pork samples (Factory A; pH 5.56). Results of the Student’s T-test did not show any significant differences between the different maturation times, but were observed between Factories A and E as the ANOVA analysis results show.

With respect to Aw, a decrease in all the samples and values between 0.957 and 0.870 were reached after half of the curing period and between 0.909 and 0.834 at the end. Furthermore, ANOVA demonstrated that there were significant differences among all samples and the same for the Student’s T-test between samples at 30 and 60 days of maturation especially in Factory E. The statistical analysis from NaCl assays also showed the significant differences in the same way. 

Physicochemical characteristics could be another factor involved in cell fluctuation throughout the drying period. Wild boar fermented sausages had the lowest pH and Aw values, and these were also the samples with the lowest counts (3.6 CFU log). Fernández-López et al. [10] reported that the lowest pH and Aw and yeast counts were reached at the end of the ripening process.

### 3.3. Yeast Identification

#### 3.3.1. Species Level

**Figure 2:**
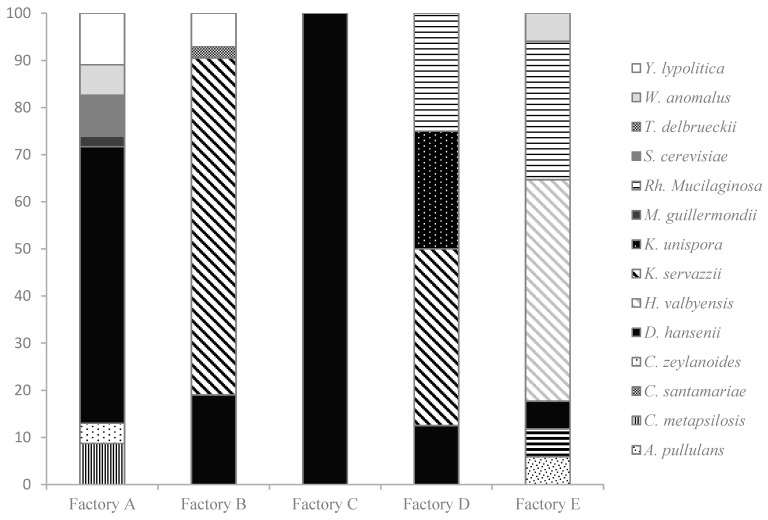
Distribution (%) of the yeast species found in the different curing rooms studied. Factories A and B: Pork meat products and C–E game meat products.

A total of 123 yeast cultures were isolated, 88 from pork sausages (A and B), and 35 from game sausages (C, D, and E). The 88 isolated yeasts from pork were classified in nine different species by sequencing the ITS region. The most isolated species were *Debaryomyces hansenii*, (35) followed by *Kazachstania servazzii* (30), which were also the majority of the species in Factories A and B, respectively. The other species identified presented less than 10 isolated species (Figure 2). Only two species, *D. hansenii* and *Yarrowia lypolitica*, were characterized in both pork factories.

Game meat presented a higher species diversity than the pork products, since 35 isolates were included in eight different species. In that case, *D. hansenii* (12) was again the most isolated species followed by *Hanseniaspora valbyensis* (8) and *Rhodotorula mucilaginosa* (7), while the rest of the species presented less than five isolates (Figure 1).

The curing rooms environments and location have an essential influence on the yeast species variation as well as physiochemical characteristics, which are the reasons why the yeast community of these fermented sausages are different to one another, although the existing predominant species could adapt easily to this type of food.

*D. hansenii* is one of the most widely distributed yeast species in fermented foods owing to its high flexibility and use of carbon and nitrogen sources. Moreover, it contributes to the sensory characteristics [9]. Other species, such as *Y. lypolitica*, *Saccharomyces cerevisiae*, *Candida zeylanoydes*, and *Rh. mucilaginosa*, have been documented in meat products [2]. *Y. lypolitica* contributes to the aroma formation due to its proteolytic and lipolytic activity. On the other hand, there is no information on the presence of the rest of the species identified, although it is known that *Candida* sp. is also frequently found in dry fermented sausages [9]. 

Lastly, a variance on the species was observed at the different curing times (data not shown). *D. hansenii* was found in Factories A and B throughout the curing process, as well as *Candida metapsilopsis* and *S. cerevisiae* (Factory A). However, *Y. lypolitica*, which was present in both Factories A and B, was only identified at the beginning of the maturation step. Regarding the game meat samples, only *D. hansenii* (Factory C) and *H. valbyensis* (Factory E) were isolated at the two different curing times and the other species were identified mostly in the samples collected at the end of the ripening process.

#### 3.3.2. Strain Level

The identification at the strain level is collected in Table 2. PCR-RAPD showed that in pork meat there were 26 different profiles from 88 isolates (29%). Regarding each species, *K. servazzii* from Factory B presented a low number of strains in comparison to its isolates, unlike *C. metapsilosis* in pork products.

In contrast, the 35 isolates from game meat products were classified into 20 different strains with a higher biodiversity (57%) than pork samples (29%). The species such as *A. pullulans*, *C. santamariae*, *D. hansenii*, *K. servazzii*, and *Wickerhamomyces anomalus* showed the highest number of strains identified, while *Rh. mucilaginosa* showed the lowest one. A high genetic variability is desirable owing to the studied biotechnological capacities, which are strain-dependent. Therefore, the highest number of strains identified, the better the biotechnological study.

In general, it has been noted that a greater number of strains have been identified from game meat in comparison with the small number of yeast isolates and species identified. Referring to the factory results, in Factory A, seven species and 18 strains were identified and in Factory E a total of six species and 10 strains were observed, with these two curing rooms having the best yeast variability.

### 3.4. Antioxidant Capability

**Figure 3:**
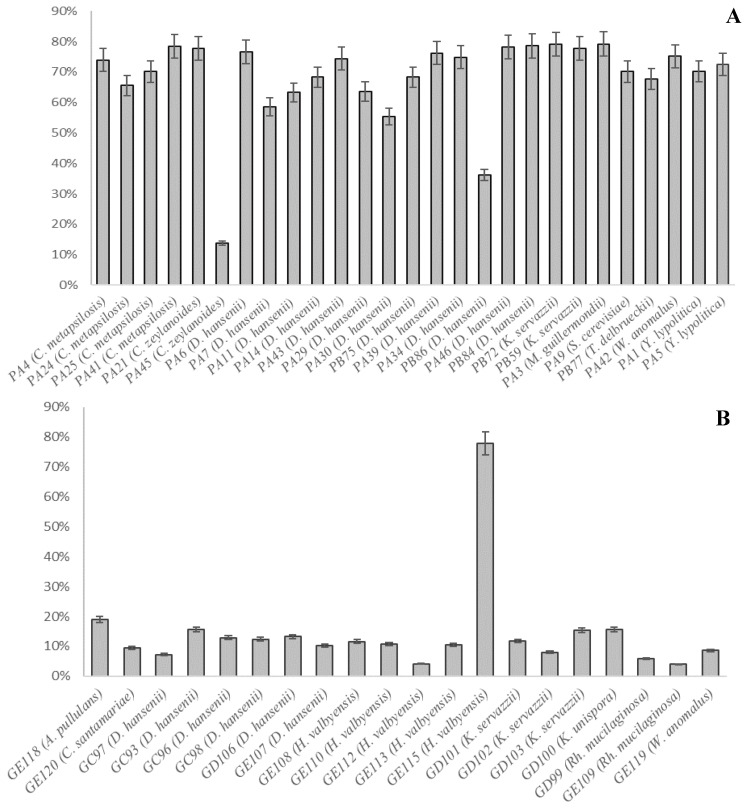
Antioxidant capability (%) of yeast strains isolated from pork meat (**A**) and game meat (**B**) products, according to the DPPH assay.

The results are expressed as an antioxidant capability percentage. In general, strains from pork fermented sausages presented higher values than those isolated from game meat products, as observed in Figure 3. 

The percentages varied between 4% (GE112) and 79% (PA3, PB72, and PB84). Strains from curing rooms A and B showed a similar behavior, except for *C. zeylanoides* PA45 and *D. hansenii* PB86, which presented the lowest antioxidant activities (14% and 36%, respectively). Nevertheless, the strains from game meat did not surpass a 20% antioxidant capacity, except H. valbyensis GE115, with a value of 78%. 

Different activities were observed in strains belonging to the same species such as *D. hansenii* and *H. valbyensis*, which indicated that the antioxidant activity is strain dependent, according to Ciafardini and Zullo [11]. 

This capability has not been studied previously in some of the tested species. Nevertheless, certain W. anomalus strains have shown a good antioxidant activity (49–51%) and have proven to be effective in avoiding olive oil oxidation, as well as some Candida sp. but not as good as the data provided by the strains tested in this study [11]. 

Few studies have been carried out on the antioxidant activity of yeast cultures in fermented meat products. However, it is known that yeasts involved in the fermentation process have an increment on this activity after the fermentation process, as Comuzzo et al. [12] documented in the wine yeast.

### 3.5. Anti-Lipid Peroxidation Capacity

**Figure 4:**
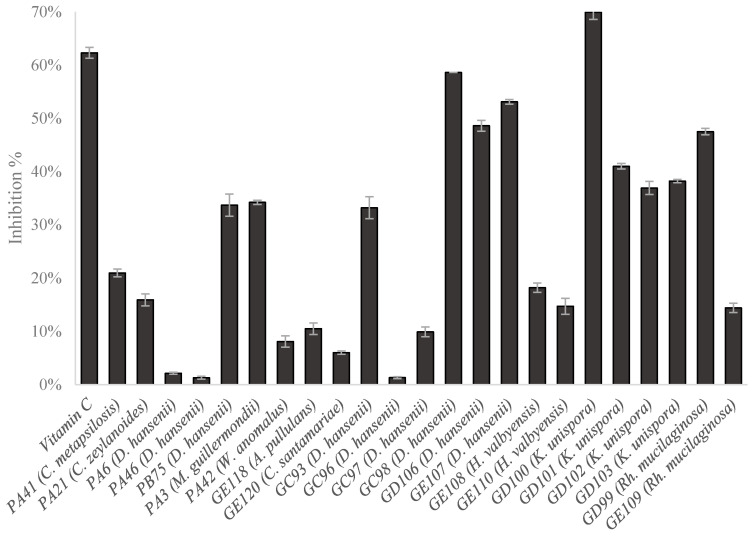
Lipid peroxidation (% inhibition) of the yeast strains studied and positive control (Vitamin D). Strains with code PA means yeasts strains from factory A (Pork), while GC, GD, GE codes indicated strains from game meat samples from factory C (GC), D (GD) and E (GD).

Results on the inhibition of lipid peroxidation ranged between 0% (no inhibition) and 100% (total inhibition). Figure 4 only shows the strains with the activity (50% of the total strains tested). 

In general, 25% of the strains from pork fermented sausages presented this capacity, while around 80% was shown in game meat sausages. Only one strain (*K. unispora* GD100) showed 69.9%, which was higher than the positive control (62.3%). As occurred in the antioxidant activity assay, the anti-lipid peroxidation capacity has turned to be strain dependent.

Furthermore, it is known that the cell wall polysaccharide fraction can inhibit the lipid peroxidation. Some studies showed that the anti-lipid peroxidation increased rapidly when the cell wall polysaccharides concentration was up to 3.2 mg/mL (β-glucans) and 1.6 mg/mL (mannans) [8,13]. Additionally, these authors found that the inhibition percentage of the lipid peroxidation did not reach 40%, which is lower than those values found in this research. Therefore, wild yeasts may be a new useful tool in food protection.

### 3.6. Inhibition Assay on Mycotoxigenic Molds

Of the 26 strains from pork products, only 11 presented a slight activity against *F. graminearum* and/or *P. crustosum*, as observed in Table 3.

From these strains, nine were capable of inhibiting *F. graminearum*, whereas only three inhibited *P. crustosum*. Additionally, only one of the strains presented an activity against both fungi (*D. hansenii* PA45), while none of them had this capability against *A. parasiticus*. 

Regarding strains from game meat products, eight strains showed a biocontrol activity against at least one mold, although only three strains showed a percentage of radius inhibition higher than 40% (Table 3). Aureobasidium pullulans GE118 and Candida santamariae GE120 inhibited almost 50% of the *A. parasiticus* mycelium. Some strains presented a slight biocontrol activity against the other two molds, but *Rh. mucilaginosa* (GE109) inhibited the *P. crustosum* growth at almost 60%. 

*D. hansenii* and *W. anomalus* have previously shown a biocontrol capability against molds due to their zymocines production [3,14]. Another pathway for growth inhibition, used by *A. pullulans*, is the production of β-glucanases or quitinases [15]. Li et al. [16] have reported that *Rh. mucilaginosa* strains were able to reduce the blue and grey mold decay on fruits.

### 3.7. Aroma Formation by Yeasts Isolated from Pork Fermented Cured Sausages and Fermented Cured Sausages from Game Meat

#### 3.7.1. Olfactory Sensorial Aroma Screening

After 10 days of incubation in YNB + 2-methylbutanoic acid + ethanol/methanol at 30 °C, the olfactory sensory evaluation permitted the detection of different aromatic descriptors. From the inoculated strains, 71% were identified as unpleasant aroma producers (sulphur, sweat, mature cheese, or boiled vegetables). Nevertheless, fruit and floral aromas were also found in some strains such as *D. hansenii*, *C. zeylanoides*, *C. metapsilosis*, and *S. cerevisiae* from pork and *D. hansenii* and *K. servazzii* from game meat (Table 4). The presence of these aromatic descriptors was associated in most cases with media supplemented with ethanol. Only *K. servazzi* GD101 formed aromatic herbs and floral aroma in media supplemented with methanol. 

*D. hansenii* is used as a culture starter in the fermentation process of sausages due to its contribution to organoleptic characteristics [17,18]. Additionally, other studies have indicated that *C. zeylanoides* and *H. valbyensis* strains also contribute to the aroma formation in dry-cured meat [19].

#### 3.7.2. Volatile Compound Formation (GC-MS)

The volatile compound production was studied in those strains that showed good olfactory properties together with the *K. servazzii* strain (GD102), which produced an unpleasant aroma. The results were expressed as the % area with respect to the total area quantified.

In Table 5, the 54 volatile compounds identified can be observed. They corresponded to esters (13), aldehydes (11), acids (11), alcohols (8), hydrocarbons (4), ketones (2), furans (2), sulphur derivates (1), and “other compounds” (the remaining volatile). The 2-methylbutanoic acid and ethanol were detected since they were added, but methanol was not detected due to its high volatility. 

Some compounds were not found in the controls (ethanol and methanol without yeasts), therefore, these must come from the cellular metabolism. Other studies have shown that aldehydes, alcohols, and esters contribute significantly to the flavor of fermented meat products [20], while other compounds such as acids and ketones may also affect and modulate the final flavor of these products.

Compounds related to lipid oxidation, e.g., aldehydes were significantly higher (*p* < 0.05) in controls than inoculated samples. Only *K. servazzi* GD102 showed similar values to the control with methanol and significantly higher with ethanol in several aldehydes. Aldehydes are strongly related to the lipid oxidation in meat products and they give fatty, rancid, or floral notes depending on their concentrations [17]. 

Additionally, ketones were significantly different in samples inoculated with methanol. 

The 2-methyl-1-butanol, 3-methyl-1-butanol, and phenylethyl alcohol only appeared in inoculated samples, as in other studies in which *D. hansenii* was inoculated [9]. 

It is known that the esters can modulate the global flavor of dry-cured meat products due to their low odor thresholds and they impart fruity notes and mask rancid odors. Likewise, the presence of esters together with aldehydes has been related to a “ripened flavor” in cured meat products [20]. Although several esters were detected in inoculated samples (Table 5), ethyl acetate was the most abundant one, as observed by Cano-García et al. [18]. *K. servazzi* GD102 showed the highest concentration of ethyl acetate (47.59%), and as a result, glue notes were detected (Table 4). 

A wide variety of acids were observed. Acetic acid was produced in larger quantities by *C. zeylanoides* (PA45), *S. cerevisiae* (PA9) and, in particular, *K. servazzi* (GD102) in comparison with other acids. 

Other compounds were detected in low concentrations. Aromatic hydrocarbons, such as ethylbenzene or 1-methyl-4-(1-methylethyl)benzene are generated in meat products [17], but they contribute little to the flavor due to their high threshold of detection. 3-(Methylthio)-1-propanol was the only sulphur compound detected and this was only produced by PA9 (*S. cerevisiae*). With respect to furans, which are normally associated with meat products, furfural was produced in all samples in contrast to 2-pentylfuran [20].

## 4. Conclusions

In conclusion, the species changed during the maturation process, nevertheless, in each curing room a dominant species was found, such as *D. hansenii* and *K. servazii* in pork products or *D. hansenii* and *H. valbyensis* in game meat products. A higher number of strains per isolated yeast was observed in the game curing rooms.

With respect to technological properties, yeasts from game meat showed better anti-lipid peroxidation and biocontrol capabilities against *A. parasiticus* and *P. crustrosum*, while the antioxidant activity and biocontrol capability against *F. graminearum* were better in strains from pork meat.

Regarding flavor improvement, almost 30% of the strains produced pleasant aroma descriptors, which were related to the meat maturation process such as esters, aldehydes, or fusel alcohols. Volatile compounds with a good sensorial impact were observed in *D. hansenii*, *C. zeylanoides*, and *S. cerevisiae* strains.

Finally, future research may aid in the studying of a possible application of some *D. hansenii* strains (GC98, PA45, GC93) as potential starter cultures in fermented pork or game meat sausages, since they could contribute to the technical and sensory improvement of the quality of these products.

## Figures and Tables

**Table 1:** Physical-chemical parameters of the studied pork and game fermented sausages.

		pH		Aw		NaCl (g/100g)	
	Factory	Curing Period		Curing Period		Curing Period	
		30 Days	60 Days	30 Days	60 Days	30 Days	60 Days
Pork meat	A	5.81 ^c^ ± 0.01	5.56 ^b^^,c^ ± 0.00	0.910 ^b^ ± 0.00	0.895 ^c^ ± 0.01 *	3.96 ^c^ ± 0.08	4.06 ^c^^,d^ ± 0.16
	B	5.29 ^b^ ± 0.01	5.22 ^b^ ± 0.04	0.939 ^a^ ± 0.00	0.909 ^b^ ± 0.01 *	2.78 ^a^ ± 0.00	3.10 ^b^ ± 0.00 *
Deer meat	C	5.26 ^b^ ± 0.01	5.14 ^a^^,b^ ± 0.01	0.873 ^c^ ± 0.00	0.857 ^d^ ± 0.00 *	3.43 ^b^ ± 0.02	3.70 ^c^ ± 0.01 *
Wild boar meat	D	5.27 ^b^ ± 0.07	5.12 ^a^^,b^ ± 0.20	0.870 ^c^ ± 0.00	0.846 ^d^ ± 0.00 *	3.42 ^b^ ± 0.01	3.71 ^c^ ± 0.00 *
	E	5.18 ^a^^,b^ ± 0.01	5.02 ^a^ ± 0.02	0.957 ^a^ ± 0.00	0.834 ^d^ ± 0.03 *	2.34 ^a^ ± 0.01	3.37 ^b^ ± 0.02 *

The different letters of the superscript indicate significant differences (*p* ≤ 0.05) between factories for the same physicochemical parameter. * Shows significant differences (*p* ≤ 0.05), based on the Student’s T-test, between samples at 30 and 60 days of curing time for the same factory.

**Table 2:** Distribution of the isolates and strains found in pork (Factories A and B) and game (Factories C, D, and E) fermented sausages.

**Species**	**Factory**	**No. of Yeast Isolates**	**No. of Strains**	**Biodiversity %**
*C. metapsilosis*	A	4	3	75
*C. zeylanoides*	A	2	2	100
*D. hansenii*	A	27	9	33.3
	B	8	4	50
*K. servazzii*	B	30	2	6.6
*M. guillermondii*	A	1	1	100
*S. cerevisiae*	A	4	1	25
*T. delbrueckii*	B	1	1	100
*Y. lypolitica*	A	5	1	20
	B	3	1	33.3
*W. anomalus*	A	3	1	33.3
**Species**	**Factory**	**No. of Isolates**	**No. of Strains**	**Biodiversity %**
*A. pullulans*	E	1	1	100
*C. santamariae*	E	1	1	100
*D. hansenii*	C	10	4	40
	D	1	1	100
	E	1	1	100
*H. valbyensis*	E	8	5	62.5
*K. servazzii*	D	3	3	100
*K. unispora*	D	2	1	50
*Rh. mucilaginosa*	D	2	1	50
	E	5	1	20
*W. anomalus*	E	1	1	100

**Table 3:** Yeast strains biocontrol activity: Inhibition % of the mycelium growth radius of mycotoxigenic molds.

	Yeast Strains	% Mycelium Radius Inhibition		
		*A. parasiticus*	*F. graminearum*	*P. crustrosum*
**Fermented cured sausages from pork meat**	*C. metapsilosis* (PA4)	-	6.7	-
	*C. metapsilosis* (PA25)	-	9.9	-
	*C. metapsilosis* (PA41)	-	6.3	-
	*D. hansenii* (PA30)	-	4.1	-
	*D. hansenii* (PA45)	-	4.1	11.1
	*D. hansenii* (PA43)	-	15.4	-
	*D. hansenii* (PB86)	-	-	17.3
	*K. servazzii* (PB72)	-	-	7.8
	*M. guillermondii* (PA3)	-	3.6	-
	*S. cerevisiae* (PA9)	-	5.3	-
	*W. anomalus* (PA42)	-	10.3	-
**Fermented cured sausages from game meat**	*A. pullulans* (GE118)	43.4	-	-
	*C. santamariae* (GE120)	48.9	-	-
	*D. hansenii* (GD106)	-	12.4	-
	*H. valbyensis* (GE108)	-	5.1	-
	*H. valbyensis* (GE115)	-	-	28.7
	*Rh. mucilaginosa* (GD99)	-	9.4	-
	*Rh. mucilaginosa* (GE109)	-	3.8	59.0
	*W. anomalus* (GE119)	5.0	-	-

**Table 4:** Olfactory sensory descriptors found by the taster in some of the yeast strains studied. Those strains selected for gas chromatography (GC) analysis are marked with *.

	Yeast Strains	Aromatic Descriptors
**Fermented cured sausages from pork meat**	*C. zeylanoides* (PA45) *	Fruity
	*C. metapsilosis* (PA4) *	Fruity
	*D. hansenii* (PA6) *	Fruity; Floral
	*D. hansenii* (PA14)	Fruity; Sweet
	*D. hansenii* (PA30)	Floral; Sweet
	*S. cerevisiae* (PA9) *	Fruity
**Fermented cured sausages from game meat**	*D. hansenii* (GC93) *	Fruity; Floral; Sweet
	*D. hansenii* (GC97)	Fruity; Sweet; Herbaceous
	*D. hansenii* (GC98)	Fruity; Sweet
	*D. hansenii* (GD106)	Fruity; Floral; Sweet
	*H. valbyensis* (GE108) *	Fruity; Sweet
	*K. servazzi* (GD101) *	Floral; Aromatics herbs (M)
	K. servazzi (GD102: Unpleasant aromas) *	Glue
		Sulphur; Sweat; Mature cheese (M)

**Table 5:** Volatile compounds quantified (mean ± standard deviation) in the headspace of samples with ethanol or methanol after incubation with different strains of yeasts (expressed in percentage of the area with respect to the total area quantified multiplied per 100).

Compound	Control E^1^	Control M ^2^	PA6 E	PA45 E	PA4 E	PA9 E	GC93 E	GE108 E	GD101 M	GD102 E	GD102 M	*p*
**Aldehydes**												
2,2-dimethyl-propanal	Nd^a^	0.35 ^b^ ± 0.02	Nd ^a^	Nd ^a^	Nd ^a^	Nd ^a^	Nd ^a^	Nd ^a^	Nd ^a^	Nd ^a^	Nd ^a^	*
Hexanal	0.31 ^a^ ± 0.17	0.72 ^b^ ± 0.05	0.23 ^a^ ± 0.08	0.10 ^a^ ± 0.03	0.23 ^a^ ± 0.07	0.13 ^a^ ± 0.12	0.28 ^a^ ± 0.07	0.12 ^a^ ± 0.02	0.07 ^a^ ± 0.04	0.07 ^a^ ± 0.01	0.79 ^b^ ± 0.17	*
Heptanal	0.09 ^a^ ± 0.07	0.19 ^a^ ± 0.04	0.05 ^a^ ± 0.01	Nd ^a^	0.05 ^a^ ± 0.01	0.03 ^a^ ± 0.02	0.14 ^a^ ± 0.01	0.05 ^a^ ± 0.02	0.09 ^a^ ± 0.03	0.03 ^a^ ± 0.01	0.43 ^b^ ± 0.26	*
Octanal	0.04 ^a^ ± 0.03	0.31 ^b^ ± 0.04	0.08 ^a^ ± 0.00	0.08 ^a^ ± 0.01	0.09 ^a^ ± 0.01	0.04 ^a^ ± 0.01	0.12 ^a^ ± 0.09	0.06 ^a^ ± 0.00	Nd ^a^	0.03 ^a^ ± 0.02	0.29 ^b^ ± 0.00	*
Nonanal	1.05 ^a,b^ ± 0.90	1.66 ^b^ ± 0.19	0.41 ^a,b^ ± 0.14	0.51 ^a,b^ ± 0.41	0.76 ^a,b^ ± 0.04	0.17 ^a^ ± 0.04	0.56 ^a,b^ ± 0.44	0.35 ^a^ ± 0.10	0.40 ^a,b^ ± 0.07	0.21 ^a^ ± 0.14	0.83 ^a,b^ ± 0.25	*
Decanal	0.56 ± 0.60	0.57 ± 0.16	0.22 ± 0.02	0.18 ± 0.04	0.23 ± 0.11	0.11 ± 0.02	0.17 ± 0.05	0.31 ± 0.01	0.30 ± 0.01	0.20 ± 0.11	0.75 ± 0.08	ns
Benzaldehyde	0.30 ^a,b^ ± 0.13	0.94 ^b^ ± 0.38	0.36 ^a,b^ ± 0.07	0.22 ^a,b^ ± 0.00	0.23 ^a,b^ ± 0.08	0.15 ^a^ ± 0.06	0.20 ^a,b^ ± 0.03	0.23 ^a,b^ ± 0.01	0.42 ^a,b^ ± 0.01	0.20 ^a,b^ ± 0.01	0.98 ^b^ ± 0.53	*
Undecanal	0.01 ^a^ ± 0.01	0.13 ^b^ ± 0.04	Nd ^a^	Nd ^a^	Nd ^a^	Nd ^a^	Nd^a^	Nd ^a^	Nd ^a^	Nd ^a^	Nd ^a^	*
Dodecanal	0.12 ^a^ ± 0.08	0.22 ^b^ ± 0.01	0.07 ^a^ ± 0.01	0.05 ^a^ ± 0.00	0.06 ^a^ ± 0.04	0.05 ^a^ ± 0.00	0.05 ^a^ ± 0.00	0.06 ^a^ ± 0.01	0.03 ^a^ ± 0.01	0.04 ^a^ ± 0.01	0.28 ^b^ ± 0.03	*
4-ethyl benzaldehyde	0.15 ^a^ ± 0.12	0.33 ^b^ ± 0.01	0.11 ^a^ ± 0.01	0.06 ^a^ ± 0.00	0.08 ^a^ ± 0.01	0.09 ^a^ ± 0.01	0.09 ^a^ ± 0.00	0.11 ^a^ ± 0.01	0.03 ^a^ ± 0.01	0.06 ^a^ ± 0.01	0.52 ^c^ ± 0.18	*
3-phenyl-2-propenal	0.10 ^a^ ± 0.01	0.26 ^b^ ± 0.14	0.07 ^a^ ± 0.02	0.04 ^a^ ± 0.01	0.05 ^a^ ± 0.01	0.04 ^a^ ± 0.01	Nd ^a^	0.05 ^a^ ± 0.00	0.15 ^a,b^ ± 0.00	0.03 ^a^ ± 0.00	0.28 ^b^ ± 0.13	*
**Ketones**												
6-methyl-5-hepten-2-one	0.10 ^a,b^ ± 0.07	0.14 ^a,b,c^ ± 0.01	0.04 ^a^ ± 0.01	0.04 ^a^ ± 0.01	0.02 ^a^ ± 0.01	0.02 ^a^ ± 0.00	0.06 ^a^ ± 0.00	0.08 ^a,b^ ± 0.03	0.19 ^b,c^ ± 0.05	0.03^a^ ± 0.00	0.22 ^c^ ± 0.09	*
Butyrolactone	0.08 ^a^ ± 0.01	0.23 ^b^ ± 0.13	0.06 ^a^ ± 0.00	0.03 ^a^ ± 0.00	0.03 ^a^ ± 0.01	0.03 ^a^ ± 0.01	0.03 ^a^ ± 0.00	0.03 ^a^ ± 0.00	0.07 ^a^ ± 0.01	0.02 ^a^ ± 0.00	0.14 ^a,b^ ± 0.02	*
**Alcohols**												
Ethanol	71.68 ^d^ ± 5.53	Nd ^a^	71.72 ^d^ ± 8.71	66.63 ^c,d^ ± 3.36	73.25 ^d^ ± 5.40	55.09 ^c^ ± 6.54	70.40 ^d^ ± 0.84	63.45 ^c,d^ ± 5.25	Nd ^a^	27.70 ^b^ ± 0.26	Nd ^a^	*
2-methoxy ethanol	Tr	0.20 ± 0.20	Nd	Nd	Nd	Nd	Nd	Nd	Nd	Nd	0.14 ± 0.18	ns
2-methyl-1-butanol	Nd ^a^	Nd ^a^	1.00 ^b^ ± 0.23	0.09 ^a^ ± 0.03	0.07 ^a^ ± 0.01	0.13 ^a^ ± 0.03	0.07 ^a^ ± 0.04	0.11 ^a^ ± 0.05	0.14 ^a^ ± 0.05	0.08 ^a^ ± 0.01	Nd ^a^	*
3-methyl-1-Butanol	Nd ^a^	Nd ^a^	2.17 ^b^ ± 0.45	0.54 ^a^ ± 0.01	0.27 ^a^ ± 0.02	0.32 ^a^ ± 0.06	0.16 ^a^ ± 0.05	0.28 ^a^ ± 0.06	0.48 ^a^ ± 0.05	0.21 ^a^ ± 0.01	Nd ^a^	*
2-ethoxy ethanol	0.01^a^ ± 0.02	0.12^b^ ± 0.03	0.04 ^a^ ± 0.01	0.04 ^a^ ± 0.01	0.04 ^a^ ± 0.02	0.03 ^a^ ± 0.02	0.02 ^a^ ± 0.01	0.03 ^a^ ± 0.00	Nd ^a^	0.02 ^a^ ± 0.01	0.23 ^c^ ± 0.01	*
1-Octanol	0.11^ab^ ± 0.08	0.23^b^ ± 0.07	0.05 ^a^ ± 0.02	0.05 ^a^ ± 0.01	0.06 ^a^ ± 0.01	0.04 ^a^ ± 0.02	0.07 ^a^ ± 0.03	0.05 ^a^ ± 0.02	0.14 ^a,b^ ± 0.05	0.02 ^a^ ± 0.00	0.34 ^c^ ± 0.08	*
2-(2-ethoxyethoxy) ethanol	0.11 ^a^ ± 0.07	0.49^b^ ± 0.19	0.15 ^a^ ± 0.03	0.10 ^a^ ± 0.02	0.11 ^a^ ± 0.05	0.08 ^a^ ± 0.02	0.11 ^a^ ± 0.01	0.11 ^a^ ± 0.02	0.25 ^a^ ± 0.09	0.07 ^a^ ± 0.01	0.51 ^b^ ± 0.20	*
Phenylethyl alcohol	Nd ^a^	Nd ^a^	1.15 ^a^ ± 0.25	1.14 ^a^ ± 0.05	0.14 ^a^ ± 0.03	0.89 ^a^ ± 0.14	0.09 ^a^ ± 0.01	0.12 ^a^ ± 0.00	0.11 ^a^ ± 0.00	0.15 ^a^ ± 0.00	5.90 ^b^ ± 1.28	*
**Ester compounds**												
Methyl acetate	Nd ^a^	Nd ^a^	Nd ^a^	Nd ^a^	Nd ^a^	Nd ^a^	Nd ^a^	Nd ^a^	0.10 ^b^ ± 0.00	Nd ^a^	Nd ^a^	*
Ethyl acetate	Nd ^a^	Nd ^a^	1.10 ^a^ ± 0.39	0.53 ^a^ ± 0.24	Nd ^a^	3.66 ^b^ ± 1.13	0.12 ^a^ ± 0.01	0.95 ^a^ ± 0.00	0.14 ^a^ ± 0.08	47.59 ^c^ ± 0.55	4.08 ^b^ ± 2.45	*
2-methyl-methyl butanoate	Nd ^a^	Nd ^a^	Nd ^a^	Nd ^a^	Nd ^a^	Nd ^a^	Nd ^a^	Nd ^a^	4.50 ^b^ ± 1.22	Nd ^a^	9.16 ^c^ ± 1.99	*
2-methyl-ethyl butanoate	0.05 ^a^ ± 0.05	Nd ^a^	0.06 ^a^ ± 0.01	0.50 ^c^ ± 0.02	0.28 ^b^ ± 0.04	0.47 ^c^ ± 0.08	0.15 ^a^ ± 0.09	0.05 ^a^ ± 0.04	Nd ^a^	0.13 ^a^ ± 0.01	Nd ^a^	*
1-butanol-3-methyl-acetate	Nd ^a^	Nd ^a^	Nd ^a^	Nd ^a^	Nd ^a^	Nd ^a^	Nd ^a^	Nd ^a^	Nd ^a^	0.02 ^b^ ± 0.00	Nd ^a^	*
3-methyl-methyl-2-butenoate	Nd ^a^	Nd ^a^	Nd ^a^	Nd ^a^	Nd ^a^	Nd ^a^	Nd ^a^	Nd ^a^	Nd ^a^	Nd ^a^	1.16 ^b^± 0.35	*
Ethyl hexanoate	Tr ^a^	0.07 ^b^ ± 0.05	Tr ^a^	Tr ^a^	Nd ^a^	Nd ^a^	Tr ^a^	Nd ^a^	Nd ^a^	Tr ^a^	Tr ^a^	*
Phenyl benzoate	Nd	0.02 ± 0.02	0.09 ± 0.07	Nd	Nd	0.07 ± 0.04	0.21 ± 0.26	0.10 ± 0.07	Nd	0.04 ± 0.00	Nd	ns
Methyl octanoate	nd	0.24 ± 0.25	Nd	nd	nd	nd	nd	nd	nd	nd	nd	ns
Ethyl nonanoate	0.02 ^a^ ± 0.01	0.27 ^b^ ± 0.09	0.13 ^a^ ± 0.04	0.11 ^a^ ± 0.02	0.13 ^a^ ± 0.05	0.06 ^a^ ± 0.05	0.09 ^a^ ± 0.00	0.10 ^a^ ± 0.00	0.10 ^a^ ± 0.01	0.06 ^a^ ± 0.03	0.02 ^a^ ± 0.00	*
Ethyl decanoate	0.06 ^a^ ± 0.05	0.18 ^b^ ± 0.08	0.07 ^a^ ± 0.02	0.04 ^a^ ± 0.00	0.05 ^a^ ± 0.02	0.05 ^a^ ± 0.00	0.06 ^a^ ± 0.00	0.05 ^a^ ± 0.00	0.02 ^a^ ± 0.01	0.03 ^a^ ± 0.02	0.19 ^b^ ± 0.03	*
1-phenylethyl acetate	Nd ^a^	Nd ^a^	0.86 ^b^ ± 0.24	0.14 ^a^ ± 0.02	0.09 ^a^ ± 0.01	0.08 ^a^ ± 0.00	0.01 ^a^ ± 0.00	0.01 ^a^ ± 0.00	Nd ^a^	0.07 ^a^ ± 0.02	Nd ^a^	*
Ethyl undecanoate	0.01 ^a^ ± 0.00	0.08 ^a,b^ ± 0.03	0.04 ^a^ ± 0.02	0.02 ^a^ ± 0.01	0.09 ^a,b^ ± 0.10	0.03 ^a^ ± 0.00	0.05 ^a^ ± 0.00	0.04 ^a^ ± 0.00	0.02 ^a^ ± 0.02	0.02 ^a^ ± 0.00	0.16 ^b^ ± 0.04	*
**Acids**												
Acetic acid	0.14 ^a^ ± 0.06	0.42 ^a^ ± 0.00	0.21 ^a^ ± 0.16	1.41 ^b^ ± 0.00	0.24 ^a^ ± 0.03	1.48 ^b^ ± 0.26	0.12 ^a^ ± 0.07	0.23 ^a^ ± 0.06	0.25 ^a^ ± 0.06	1.96 ^c^ ± 0.00	0.47 ^a^ ± 0.33	*
Propanoic acid	0.01 ^a^ ± 0.00	0.14 ^b^ ± 0.04	0.02 ^a^ ± 0.01	0.05 ^a^ ± 0.01	0.01 ^a^ ± 0.01	0.04 ^a^ ± 0.02	0.05 ^a^ ± 0.02	0.06 ^a^ ± 0.01	Nd ^a^	0.04 ^a^ ± 0.00	0.03 ^a^ ± 0.01	*
Butanoic acid	0.08 ^a^ ± 0.05	0.32 ^b^ ± 0.05	0.13 ^a^ ± 0.04	0.09 ^a^ ± 0.02	0.08 ^a^ ± 0.04	0.07 ^a^ ± 0.01	0.06 ^a^ ± 0.00	0.07 ^a^ ± 0.00	Nd ^a^	0.05 ^a^ ± 0.00	0.40 ^c^ ± 0.07	*
2-methyl butanoic acid	22.32 ^a,b^ ± 1.35	80.56 ^d^ ± 7.14	16.58 ^a^ ± 7.33	25.31 ^a,b^ ± 2.47	20.92 ^a,b^ ± 4.23	34.50 ^b^ ± 5.34	24.08 ^a,b^ ± 0.78	30.14 ^a,b^ ± 5.67	86.13 ^d^ ± 1.80	18.95 ^a,b^ ± 0.04	60.64 ^c^ ± 4.54	*
Pentanoic acid	0.09 ^a^ ± 0.07	0.43 ^c^ ± 0.14	0.12 ^a^ ± 0.05	0.04 ^a^ ± 0.02	0.05 ^a^ ± 0.04	0.06 ^a^ ± 0.03	0.08 ^a^ ± 0.02	0.09 ^a^ ± 0.02	0.05 ^a^ ± 0.03	0.05 ^a^ ± 0.00	0.27 ^b^ ± 0.04	*
Hexanoic acid	0.17 ^a^ ± 0.10	0.89 ^b^ ± 0.40	0.14 ^a^ ± 0.07	0.10 ^a^ ± 0.03	0.11 ^a^ ± 0.07	0.08 ^a^ ± 0.00	0.08 ^a^ ± 0.01	0.08 ^a^ ± 0.00	0.23 ^a^ ± 0.08	0.06 ^a^ ± 0.00	0.54 ^a^ ± 0.09	*
Benzoic acid	Nd ^a^	Nd ^a^	0.22 ^b^ ± 0.01	0.22 ^b^ ± 0.08	0.04 ^a^ ± 0.00	0.04 ^a^ ± 0.02	0.05 ^a^ ± 0.00	0.05 ± 0.00	0.20 ^b^ ± 0.11	0.02 ^a^ ± 0.00	Nd ^a^	*
Heptanoic acid	0.17 ^a,b^ ± 0.14	0.54 ^c^ ± 0.02	0.21 ^a,b^ ± 0.04	0.12 ^a^ ± 0.04	0.14 ^a,b^ ± 0.07	0.07 ^a^ ± 0.06	0.14 ^a,b^ ± 0.01	0.13 ^a,b^ ± 0.03	0.33 ^b^ ± 0.05	0.10 ^a^ ± 0.02	0.56 ^c^ ± 0.05	*
Octanoic acid	0.50 ^a^ ± 0.24	2.68 ^b^ ± 1.89	0.38 ^a^ ± 0.03	0.27 ^a^ ± 0.00	0.50 ^a^ ± 0.14	0.41 ^a^ ± 0.01	0.38 ^a^ ± 0.01	0.52 ^a^ ± 0.15	1.06 ^a^ ± 0.23	0.33 ^a^ ± 0.13	1.31 ^a^ ± 0.07	*
Nonanoic acid	0.70 ^a^ ± 0.43	3.02 ^a,b^ ± 1.26	0.81 ^a^ ± 0.13	0.60 ^a^ ± 0.24	0.98 ^a^ ± 0.06	0.73 ^a^ ± 0.13	0.93 ^a^ ± 0.39	0.87 ^a^ ± 0.56	2.13 ^a,b^ ± 0.14	0.78 ^a^ ± 0.07	4.11 ^b^ ± 2.44	*
Decanoic acid	0.40 ^a^ ± 0.30	1.35 ^a,b^ ± 0.75	0.43 ^a^ ± 0.11	0.25 ^a^ ± 0.03	0.31 ^a^ ± 0.05	0.34 ^a^ ± 0.11	0.40 ^a^ ± 0.05	0.42 ^a^ ± 0.03	1.34 ^a,b^ ± 0.58	0.30 ^a^ ± 0.06	1.57 ^b^ ± 0.01	*
**Aromatic hydrocarbons**												
Ethylbenzene	0.01 ± 0.00	0.13 ± 0.08	Nd	Nd	Nd	Nd	Nd	Nd	Nd	0.01 ± 0.00	0.85 ± 0.83	ns
1-ethyl-3-methyl benzene	Nd ^a^	Nd ^a^	Nd ^a^	Nd ^a^	Nd ^a^	Nd ^a^	Nd ^a^	Nd ^a^	Nd ^a^	Nd ^a^	0.19 ^b^ ± 0.05	*
1-methyl-4-(1-methylethyl)-benzene	Nd ^a^	0.06 ^a^ ± 0.02	0.09 ^a^ ± 0.07	Nd ^a^	Nd ^a^	0.07 ^a^ ± 0.04	0.21 ^b^ ± 0.26	0.10 ^a^ ± 0.07	Nd ^a^	0.04 ^a^ ± 0.00	Nd ^a^	*
Trimethylbenzene	Nd ^a^	Nd ^a^	Nd ^a^	Nd ^a^	Nd ^a^	Nd ^a^	Nd ^a^	Nd ^a^	Nd ^a^	Nd ^a^	0.22 ^b^ ± 0.15	*
**Sulphur compounds**												
3-(methylthio)-1-propanol	Nd ^a^	Nd ^a^	Nd ^a^	Nd ^a^	Nd ^a^	0.07 ^b^ ± 0.01	Nd ^a^	Nd ^a^	Nd ^a^	Nd ^a^	Nd ^a^	*
Furans												
2-pentyl furan	0.02 ^a^ ± 0.00	0.06 ^b^ ± 0.02	0.01 ^a^ ± 0.00	Tr ^a^	Nd ^a^	Nd ^a^	Tr ^a^	0.02 ^a^ ± 0.01	Nd ^a^	0.01 ^a^ ± 0.00	Nd ^a^	*
Furfural	0.08 ^a,b^ ± 0.05	0.30 ^c^ ± 0.04	0.06 ^a,b^ ± 0.01	0.05 ^a,b^ ± 0.01	0.03 ^a^ ± 0.02	0.02 ^a^ ± 0.01	0.03 ^a^ ± 0.00	0.04 ^a,b^ ± 0.00	0.08 ^a,b^ ± 0.02	0.04 ^a,b^ ± 0.01	0.18 ^b^ ± 0.10	*
**Others compounds**												
Benzonitrile	0.11 ^a,b^ ± 0.06	0.29 ^c^ ± 0.07	0.10 ^a,b^ ± 0.01	0.06 ^a^ ± 0.00	0.08 ^a,b^ ± 0.01	0.05 ^a^ ± 0.01	0.08 ^a,b^ ± 0.01	0.08 ^a,b^ ± 0.02	0.17 ^b^ ± 0.03	0.04 ^a^ ± 0.01	0.36 ^d^ ± 0.02	*
Phenol	0.24 ^a,b^ ± 0.20	0.87 ^d^ ± 0.15	0.23 ^a,b^ ± 0.08	0.20 ^a,b^ ± 0.02	0.13 ^a^ ± 0.03	0.17 ^a^ ± 0.07	0.21 ^a,b^ ± 0.07	0.28 ^a,b^ ± 0.03	0.50 ^b,c^ ± 0.09	0.11 ^a^ ± 0.01	0.70 ^c,d^ ± 0.06	*

^1^ E: Medium incubated with ethanol; ^2^ M: Medium incubated with methanol. Nd = Not detected. Tr = Trace. Superscript letters (a–d) in the same row indicate significant differences among samples; Ns = *p* > 0.05; * = *p* < 0.05.
